# Empyema associated with community-acquired pneumonia: A Pediatric Investigator's Collaborative Network on Infections in Canada (PICNIC) study

**DOI:** 10.1186/1471-2334-8-129

**Published:** 2008-09-25

**Authors:** Joanne M Langley, James D Kellner, Nataly Solomon, Joan L Robinson, Nicole Le Saux, Jane McDonald, Rolando Ulloa-Gutierrez, Ben Tan, Upton Allen, Simon Dobson, Heather Joudrey

**Affiliations:** 1 Department of Pediatrics, Dalhousie University, Halifax, Canada; 2 IWK Health Centre, Halifax, Canada; 3 Alberta Children's Hospital, Calgary, Canada; 4 University of Calgary, Calgary, Canada; 5 Stollery Children's Hospital, Edmonton, Canada; 6 University of Alberta, Edmonton, Canada; 7 Children's Hospital of Eastern Ontario, Ottawa, Canada; 8 University of Ottawa, Ottawa, Canada; 9 Montreal Children's Hospital, Montreal, Canada; 10 University of British Columbia,Vancouver, Canada; 11 Women's and Children's Hospital of British Columbia, Vancouver, Canada; 12 University of Saskatchewan, Saskatoon, Canada; 13 Hospital for Sick Children, Toronto, Canada; 14 Hospital for Sick Children, University of Toronto, Toronto, Canada

## Abstract

**Background:**

Although the incidence of serious morbidity with childhood pneumonia has decreased over time, empyema as a complication of community-acquired pneumonia continues to be an important clinical problem. We reviewed the epidemiology and clinical management of empyema at 8 pediatric hospitals in a period before the widespread implementation of universal infant heptavalent pneumococcal vaccine programs in Canada.

**Methods:**

Health records for children < 18 years admitted from 1/1/00–31/12/03 were searched for ICD-9 code 510 or ICD-10 code J869 (Empyema). Empyema was defined as at least one of: thoracentesis with microbial growth from pleural fluid, or no pleural fluid growth but compatible chemistry or cell count, or radiologist diagnosis, or diagnosis at surgery. Patients with empyemas secondary to chest trauma, thoracic surgery or esophageal rupture were excluded. Data was retrieved using a standard form with a data dictionary.

**Results:**

251 children met inclusion criteria; 51.4% were male. Most children were previously healthy and those ≤ 5 years of age comprised 57% of the cases. The median length of hospitalization was 9 days. Admissions occurred in all months but peaked in winter. Oxygen supplementation was required in 77% of children, 75% had chest tube placement and 33% were admitted to an intensive care unit. While similarity in use of pain medication, antipyretics and antimicrobial use was observed, a wide variation in number of chest radiographs and invasive procedures (thoracentesis, placement of chest tubes) was observed between centers. The most common organism found in normally sterile samples (blood, pleural fluid, lung biopsy) was *Streptococcus pneumoniae*.

**Conclusion:**

Empyema occurs most commonly in children under five years and is associated with considerable morbidity. Variation in management by center was observed. Enhanced surveillance using molecular methods could improve diagnosis and public health planning, particularly with regard to the relationship between immunization programs and the epidemiology of empyema associated with community-acquired pneumonia in children.

## Background

Although morbidity and mortality associated with community-acquired pneumonia in childhood has declined markedly in the last 60 years [1], parapneumonic empyema complicating pneumonia continues to be an important problem in North America and Europe [2-13]. The diagnostic and therapeutic approach to empyema in children is not standardized and available guidelines are largely based on consensus opinion because of inadequate evidence supporting various therapeutic interventions[14]. We report a multi-center study of parapneumonic empyema in eight Canadian children's hospitals over a three-year period to describe the epidemiology of this serious childhood infection, including microbiologic etiology and clinical presentation and management across centers. Our intent was to determine if management variation occurred across centers, and to describe the burden of illness associated with various pathogens before the anticipated widespread introduction of universal infant pneumococcal vaccine programs.

## Methods

Participating investigators from the Pediatric Investigators Collaborative Network on Infections in Canada (PICNIC) conducted the study at eight university-affiliated pediatric acute care facilities across Canada.

Possible cases were identified at each hospital from a search of health records databases to identify children under 18 years of age, admitted to hospital for at least 24 hours 1 January 2000 to 31 December 2003, and who had a diagnosis of empyema (International Classification of Diseases Clinical Modification-9 (ICD-9) code 510 or ICD-10 code J869 (Empyema).

The health record of each possible case was reviewed and included in the study if they had at least one of the following criteria: 1) thoracentesis with microbial growth from pleural fluid or 2) thoracentesis with no growth on culture of pleural fluid but elevated protein, or cell count (normal and abnormal reference values as determined by the clinical laboratory at each center) 3) ultrasound or other diagnostic imaging evidence of pleural fluid assessed by the radiologist as empyema or 4) diagnosis at time of thoracic surgery. Because our intent was to focus on empyema associated with community acquired respiratory tract infections, children with empyema associated with other illnesses such as chest trauma, thoracic surgery, or esophageal rupture were excluded.

A standardized case report form, with a data dictionary, was developed to collect data on demographic, clinical, diagnostic, treatment and outcome features of all cases. If available, ethnicity was described including whether the child was aboriginal (e.g. Indian, Inuit and Métis peoples of Canada). Tachypnea was defined by age: if < 12 months of age, > 75 breaths/minute; if > 12 months and < 10 years old, > 40 breaths/minute, if > 10 years old then > 30 breaths/minute. An elevated temperature was defined as temperature > 38.5°C. Death within 2 weeks of admission was noted. Data was entered at the coordinating site and analyzed using the software program SAS Version 8.2 (SAS Institute Inc., Cary, North Carolina).

We determined if bacterial pathogens were identified from sterile site samples including blood, pleural fluid and lung tissue at the time the child was diagnosed. For cases where *Streptococcus pneumoniae *was identified, the isolates were sent for serotyping at the National Centre for Streptococcus in Edmonton, Alberta as part of the Canadian Paediatric Society/Public Health Agency of Canada Immunization Monitoring Program Active (IMPACT) surveillance system[15]. Isolates were serotyped using the Quellung reaction, as previously described [15,16].

The study was approved by the institutional review board at each site.

## Results

### Demographics

**Figure 1:**
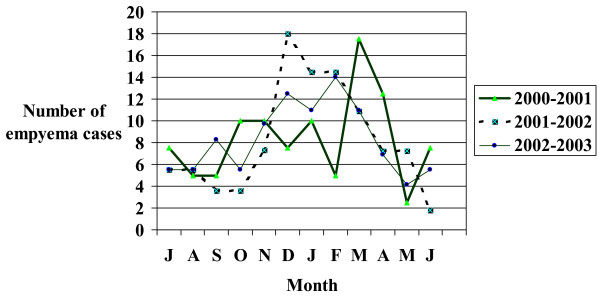
Incidence of admission with empyema associated with community-acquired pneumonia in Canadian children at 8 pediatric hospitals by month, 2001–2003.

There were 251 cases in 251 children in 8 pediatric health centers who met inclusion criteria; 51.4% were male. The average age was 6.0 years (SD +/- 4.98). Caucasians comprised 42%; aboriginals 18%, Asians 7%, African-Canadian < 1% and other 12% (no data 20%). Most children were previously healthy (78%; 195/251). One or more underlying conditions were present in 19% (51/251) of children including asthma (n = 45), congenital heart disease (n = 8), immunocompromised state (n = 5), and cystic fibrosis (n = 2). In immunocompetent children the mean age was 6.0 years (SD 5.01) and the age distribution was ≤ 1 year = 8%, 12–23 months 11%, 24–35 months 14%%, 3 to 5 years = 24%, 6–10 years = 19%, ≥ 11 years = 24%. Admissions occurred in all months of the year but peaked between November and April (Figure 1).

Aboriginal children accounted for 18% of all admissions (38/246). The mean age of these children was 4.2 years (+/-4.21 years) and 58% (22/38) were ≤ 2 years of age. 51.5% were female. Almost all of these children were healthy prior to this illness (86%; 33/38); 5 had unspecified pre-existing lung disease. Most aboriginal children were transferred to the participating pediatric facilities from their home communities (74%; 28/38) whereas fewer non-aboriginal children were admitted from a referring hospital (52.7%; 125/237).

### Microbiologic etiology

A specific microbial diagnosis was made in 32% (80/251) of children. If an organism was identified in a sterile site sample (blood, pleural fluid, lung biopsy or thoracotomy specimen) it was considered causative. Only one child had two organisms identified (*Streptococcus pneumoniae *and *Streptococcus pyogenes*). The organisms were *S. pneumoniae *(n = 38), *S. pyogenes *(n = 20), *Staphylococcus aureus *(8), *S. milleri (6), Fusobacteria *(2) and one each of *Mycobacterium tuberculosis, Eikenella corrodens *and *echinococcus*.

Among the 38 aboriginal children with positive cultures the causative organisms were *S. pneumoniae *(n = 6) and tuberculosis (n = 1).

A causative organism was identified for only one of the five immunocompromised children (*S. pneumoniae*).

Of the 35 *S. pneumoniae *isolates that were available to be typed, 48% were due to serotypes present in the 7-valent pneumococcal conjugate vaccine: serotype 14 (n = 10), 6B (n = 3), 9V (n = 2), 23F (n = 1) and 4 (n = 1). Other serotypes were 1(n = 8), 3 (n = 9), 6A (n = 1).

### Clinical course and management

**Figure 2:**
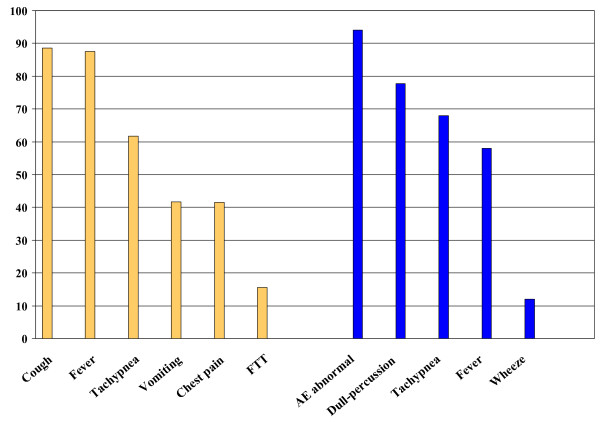
Clinical presentation of children with empyema associated with community-acquired pneumonia: percentage of children with each symptom and sign on admission.

At presentation the most common symptoms were fever, cough and breathing difficulty (tachypnea), and the most common signs were abnormal air entry on auscultation, dullness to percussion, tachypnea, and fever (Figure 2). The diagnosis of empyema was made within 24 hours of admission in 63% of children and by the fifth hospital day in 95%. The most commonly performed diagnostic procedure was chest radiography (96.8% of children). About 16% of children had at least seven chest radiographs during their stay while 10% of children had two or fewer chest radiographs. Computerized tomography of the chest was performed in 64% of children (range 25–86% of children by center). Chest ultrasound was performed on 51% of children (range 28–90% of children by center).

**Figure 3:**
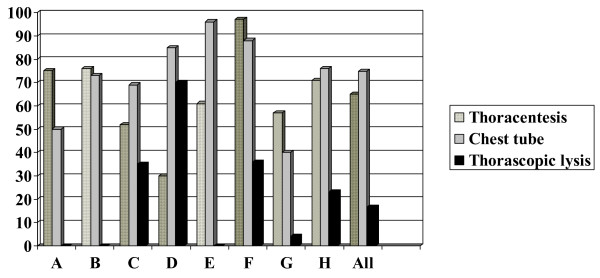
Variation in use of invasive interventions for management of children with empyema at 8 Canadian pediatric hospitals.

Although subspecialty consultation to the infectious disease, pulmonology, or surgery services was almost uniformly sought across the country (99% of all patients), use of chest tubes, thoracentesis and thorascopic lysis varied from center to center (Figure 3). Three centers did not perform thorascopic lysis during the study period. Chest tube placement occurred in 75% of children overall. The overall median time to chest tube insertion after admission was 1.5 days; all sites had a median insertion time of three days or less. The mean length of stay did not differ between children with and without chest tube placement (15.5 days v. 13.3 days, p = 0.30). Hospital stay ranged from 2 to 160 days, with a mean length of stay of 15 days (SD 15) and a median length of 9 days. The mean length of stay in immunocompromised children was 29 days (SD 15.4). No correlation was observed between length of hospital stay and the time to chest tube insertion (Pearson correlation coefficient 0.08).

The most commonly used intravenous antibiotic was a second-generation cephalosporin (cefuroxime) (159/251 children; 63%), followed by a third generation cephalosporin (cefotaxime) in 43% of children and clindamycin in 41% of children. Less frequently used antibiotics were cloxacillin (24% of children; 61/251), penicillin (21%), ceftriaxone (15%), and vancomycin (14%). Less than 10% of children received the following antibiotics: macrolide, aminoglycoside, extended spectrum penicillin, cefazolin, cotrimoxasole, other cephalosporins or monobactams, and quinolones.

Blood culture was performed in 212 of 251 children. Laboratory confirmation from blood only was made in 34/212 children and from pleural fluid only in 56/88 children. A Mantoux test was done on 41% of children (100/246); one of these was positive in a child with laboratory-confirmed infection.

All children received antipyretics and 79% received pain medication in addition. Parenteral feeding was required by 22% of patients, supplemental oxygen by 77% and admission to an intensive care unit by 33%. No significant difference in the use of these supportive modalities was seen across centers. A single death occurred within 2 weeks of admission for empyema in an aboriginal child with severe developmental delay and recurrent aspiration who was critically ill on admission.

When children with *S. aureus, S. pyogenes*, and *S. pneumoniae *who had chest tube placement were compared, those with *S. aureus *had a longer duration of fever (12.3 days v. 7 v. 4 days, p = 0.02). No significant differences in use of oxygen supplementation, admission to intensive care unit, mean time to insertion of a chest tube or duration of hospital stay were seen in children with each of these organisms.

## Discussion

Parapneumonic empyema occurs most commonly in previously healthy children under five years of age and is associated with considerable morbidity and need for institutional care. The majority of children in this study at all centers required pain medication (78%), supplemental oxygen (77%) and had placement of a chest drain (75%). Over half were transferred from their home communities to a tertiary care facility for relatively prolonged admissions, although in most children the length of stay was 9 days or less. Empyema appears to exact a disproportionate burden in aboriginal children, who comprised 18% of these patients, despite representing less than 3.3% percent of the overall Canadian population (census 2001, Statistics Canada). Over 75% of aboriginal children were transferred from their home community for acute care. Although empyema is associated with considerable morbidity and lengthy hospital stays, only one death occurred within two weeks of diagnosis. We were not able to look at long term pulmonary function but previous studies have shown that restrictive lung function patterns are seen in up to 90% of children with parapneumonic empyema three months after discharge, but normal lung function is present at one year follow-up[17].

Marked variation in patient management was observed in this multi-center study. Consultation to pulmonology, infectious diseases, and surgical services appears to be routine in all centers, but diagnostic and therapeutic interventions vary. As has been noted in a recent review by the British Thoracic Society, wide practice variation in the management of empyema is likely due to the lack of good evidence available to inform best management, which results in treatment based on physician experience and local bias[14]. Of over 50 recommendations considered in that review, only four had high quality evidence [14]. The only randomized controlled clinical trials of therapy for pediatric empyema have examined intrapleural installation of fibrinolytics compared to video-assisted thorascopy or installation of normal saline [18-20]. A number of case series suggest that this therapy is associated with increased pleural drainage and avoidance of surgery [14] but the evidence from the three randomized trials is conflicting. We cannot make conclusions about the efficacy of different interventions in this non-randomized observational study, but it is nonetheless interesting to note that no difference in length of stay was observed in children with and without chest tube placement, and that time to chest tube placement did not appear to alter length of stay. Development of consensus guidelines or critical care pathways by inter-disciplinary multicenter group could improve standardization of care until better quality data to guide decision making is available.

The most common organism isolated from parapneumonic effusions during this three-year period was *S. pneumoniae*, followed by *S. pyogenes, Staphylococcus aureus *and *S. milleri*. In contrast with most studies, we found *S. pyogenes *as important a cause of empyema as *S. aureus *[21]. Based on available serotyping data from this study some empyema is potentially vaccine-preventable by heptavalent pneumococcal conjugate vaccines (PCV7). In the large randomized controlled efficacy trial of PCV7 vaccine a 17% efficacy in the prevention of a positive chest radiograph was observed in the intent-to-treat group as was a 70% decrease in consolidative pneumonia (consolidation on CXR 2.5 cm or greater)[10,22-24]. Post-licensure surveillance indicates that invasive pneumococcal disease among children in the US has declined following universal vaccination [25]. PCV7 infant vaccine programs were not yet in place in most provinces in Canada at the beginning of the study period. In two of the six provinces infant immunization programs began 3 and 16 months, respectively, before the study ended. Thus in one province the vaccine could have prevented *S. pneumoniae *associated empyema in infants 6–18 months of age who would have been eligible for the study. At the time of writing almost all jurisdictions have implemented infant conjugate pneumococcal vaccine programs. The observation of an increasing incidence of empyema due to pneumococcus in some jurisdictions with universal vaccine programs, particularly due to strains not covered in the vaccine [2], is concerning, and highlights the importance of surveillance to detect changes in the epidemiology of disease following the implementation of vaccine programs. We plan to repeat this study following the more widespread introduction of pneumococcal vaccine in Canada.

Children with *S. aureus*-associated infection had longer length of stay than children with *S. pneumoniae *or *S. pyogenes*. Methicillin-resistant *S. aureus *(MRSA)infection was not common in Canadian children during the years of this study[26], and we did not identify this organism in our population.

Definitive microbiologic diagnosis was obtained in only 32% of children in this study and so most children were exposed to unnecessarily broad-spectrum antibiotics for many weeks. *S. pneumoniae *has been reported in over 70% of culture-negative empyema fluids[27] when molecular diagnosis is systematically used, and increased detection with 16SrDNA PCR [28]. This testing should become standard practice for care of children with culture-negative empyema in order follow the effect of universal vaccine programs on the serotype epidemiology of invasive disease. If detection of antibiotic resistant genes is included in the molecular testing, use of broad spectrum antimicrobials may be avoided. In the United Kingdom an enhanced surveillance program for pediatric empyema is planned for England and Wales which will include molecular diagnosis of culture-negative cases [5]. Given the evidence that *S. pneumoniae *causes most empyema in children in this study, and that a larger percentage may be found when rigorous diagnosis is pursued[27,28], we recommend more systematic use of molecular techniques to determine microbiologic etiology of empyema. These techniques should also be considered in the implementation of surveillance programs for invasive bacterial disease so that vaccine and non-vaccine serotypes of *S. pneumoniae *can be documented following introduction of childhood vaccine.

One limitation of this study is that it was not population-based and thus the incidence of parapneumonic empyema for the whole country cannot be determined from this data. However, each hospital is the only referral center for pediatric care in each region and therefore we likely captured all cases in the participating sites. Secondly, we did not define a specific pleural fluid white blood cell count required for the diagnosis of empyema, rather allowing each center to define normal/abnormal fluid obtained by thoracentesis. The condition of empyema has been used variably^21^, but usually denotes a complicated effusion associated with an adjacent pneumonic process, which is distinguished from a pleural effusion, and from an uncomplicated parapneumonic effusion associated with pneumonia. We expect that the search strategy for this review, extraction of all records with ICD-9 CODE 510 or the ICD-10 CODE J869 (empyema), would have minimized this misclassification error. As well, each patient was seen by infectious disease, surgical and respiratory specialist physicians who agreed with the diagnosis of empyema. However, the lack of data on the white blood cell count in the pleural fluid could mean that uncomplicated parapneumonic effusions could have been included in our sample. This misclassification would have increased the number of cases.

## Conclusion

Empyema associated with community-acquired pneumonia is an important cause of morbidity in Canadian children. Previously healthy children under five years of age are most commonly affected, and usually present in the winter months with tachypnea, fever, cough, and abnormal air entry on auscultation associated with dullness to percussion. The median length of hospital stay is 9 days; *S. aureus *infection is associated with longer hospitalization than *S. pneumoniae *or *S. pyogenes*. The most common infecting organism is *S. pneumoniae*. Considerable variation in use of chest tubes, thoracentesis and thorascopic lysis is observed across settings.

## Abbreviations

MRSA: Methicillin resistant *Staphylococcus aureus*.

## Competing interests

The authors declare that they have no competing interests.

## Authors' contributions

JML and JDK conceived the study. JML wrote the protocol and all authors except HJ, RU and NS subsequently provided intellectual input into its revision. All authors except HJ participated in data collection. JML wrote the manuscript and all authors reviewed it for important intellectual content, suggested revisions and reviewed the final draft. HJ performed the statistical analysis.

## Pre-publication history

The pre-publication history for this paper can be accessed here:


